# Patient-reported questionnaires in MS rehabilitation: responsiveness and minimal important difference of the multiple sclerosis questionnaire for physiotherapists (MSQPT)

**DOI:** 10.1186/s12883-017-0834-1

**Published:** 2017-03-16

**Authors:** Nico Arie van der Maas

**Affiliations:** Institut für Physiotherapieforschung, Lindenweg 48, 2503 Biel, Switzerland

**Keywords:** Multiple sclerosis, Rehabilitation, Physical therapy, Patient outcome assessment, Questionnaires, Responsiveness

## Abstract

**Background:**

The Multiple Sclerosis Questionnaire for Physical Therapists (MSQPT) is a patient-rated outcome questionnaire for evaluating the rehabilitation of persons with multiple sclerosis (MS). Responsiveness was evaluated, and minimal important difference (MID) estimates were calculated to provide thresholds for clinical change for four items, three sections and the total score of the MSQPT.

**Methods:**

This multicentre study used a combined distribution- and anchor-based approach with multiple anchors and multiple rating of change questions. Responsiveness was evaluated using effect size, standardized response mean (SRM), modified SRM and relative efficiency. For distribution-based MID estimates, 0.2 and 0.33 standard deviations (SD), standard error of measurement (SEM) and minimal detectable change were used_._ Triangulation of anchor- and distribution-based MID estimates provided a range of MID values for each of the four items, the three sections and the total score of the MSQPT. The MID values were tested for their sensitivity and specificity for amelioration and deterioration for each of the four items, the three sections and the total score of the MSQPT. The MID values of each item and section and of the total score with the best sensitivity and specificity were selected as thresholds for clinical change.

The outcome measures were the MSQPT, Hamburg Quality of Life Questionnaire for Multiple Sclerosis (HAQUAMS), rating of change questionnaires, Expanded Disability Status Scale, 6-metre timed walking test, Berg Balance Scale and 6-minute walking test.

**Results:**

The effect size ranged from 0.46 to 1.49. The SRM data showed comparable results. The modified SRM ranged from 0.00 to 0.60. Anchor-based MID estimates were very low and were comparable with SD- and SEM-based estimates. The MSQPT was more responsive than the HAQUAMS in detecting improvement but less responsive in finding deterioration. The best MID estimates of the items, sections and total score, expressed in percentage of their maximum score, were between 5.4% (activity) and 22% (item 10) change for improvement and between 5.7% (total score) and 22% (item 10) change for deterioration.

**Conclusions:**

The MSQPT is a responsive questionnaire with an adequate MID that may be used as threshold for change during rehabilitation of MS patients.

**Trial registration:**

This trial was retrospectively (01/24/2015) registered in ClinicalTrials.gov as NCT02346279.

**Electronic supplementary material:**

The online version of this article (doi:10.1186/s12883-017-0834-1) contains supplementary material, which is available to authorized users.

## Background

Many individuals with multiple sclerosis (MS) undergo physical therapy. Because MS cannot be cured, physical therapy focuses on preserving and increasing quality of life. The effects of treatment on the quality of life of MS patients should be measured at all relevant levels defined by the World Health Organization in the International Classification of Functioning, Disability and Health: body functions and structures, activities and participation. Patient Rated Outcome (PRO) instruments, such as the Short Form Health Survey (SF-36), the Multiple Sclerosis Impact Scale (MSIS-29) or the Hamburg Quality of Life Questionnaire in Multiple Sclerosis (HAQUAMS), can measure the influence of MS on the quality of life. However, these questionnaires do not focus specifically on the goals of physiotherapy or the effects of physiotherapeutic treatment. To enable an appropriate assessment of treatment-related improvement in chronic diseases, such as MS, and be applicable even for MS patients with slow progression, the questionnaire should be able to measure small changes in activity and participation. Furthermore, the questionnaire should contain sufficient items related to activities and participation that are important for the daily life of MS patients and that can be influenced by physiotherapy.

The Multiple Sclerosis Questionnaire for Physiotherapists (MSQPT) is a German patient-reported outcome measure (see Additional files 1 and 2) that was designed as an aid for physiotherapists to assess the course of treatment in MS patients. The MSQPT has 34 items that are related to physiotherapeutic treatment and are relevant for activities and participation that can be influenced by physiotherapy. They describe different aspects of the impact of MS on patient daily life and the impact of physiotherapeutic treatment [1, 2].

The answers are given on a 9- or 10-point scale. The former is a symmetric, bipolar, Likert-like scale that may be treated as an interval scale [3, 4], as stated and discussed in [1]. Three items (8b, 9a and 9b) have a 10-point interval scale.

Table 1 shows the range of scores of three sections of the MSQPT, the total score of the MSQPT and four reliable items. The three sections were identified by factor analysis [1]. They consist of activity-associated, participation-associated and balance items and were labelled as the activity, participation and balance sections, respectively [1]. The three sections of Table 1 and the total score of the MSQPT are reliable (Table 2). The criterion validity of the MSQPT, using the SF-36 and HAQUAMS as criteria, is high [1]. The MSQPT also fulfils additional demands for assessments (comparability, economy, usefulness and acceptance) [1, 2].

**Table 1:** Description of relevant items and sections of the Multiple Sclerosis Questionnaire for Physiotherapists

Item/Section	Description	Range of scores
4	I can take a shower by myself.	1–9
8a	How far can you walk on flat ground without sitting down?	1–9
8b	How long can you walk on flat ground without sitting down?	1–10
10	I can get in and out of the car by myself.	1–9
Activity section (14 items)	Dressing, bathing, standing, walking, climbing stairs, getting in and out of a car, using public transport, strenuous activities, writing, spasticity	14–128
Participation section (11 items)	Feeling rested, vitality, physical strength, fatigue, being active, adaption of activities, resilience, family life, going on a trip, fear of the consequences of MS	11–199
Balance section (2 items)	Taking a shower, balance	2–18
Total score (34 items)	Activity, participation and balance sections; global rating of change; brushing teeth, pain, sensitivity, bladder control, defecation; goals in life.	34–308

**Table 2:** Reliability of 4 items, all 3 sections and the total score of the Multiple Sclerosis Questionnaire for Physiotherapists [1]

Section	Reliability (Pearson’s r)
Taking a shower	0.90
How far can you walk?	0.95
How long can you walk?	0.89
Getting in and out of a car	0.80
Activity section	0.93
Participation section	0.77
Balance section	0.84
Total score	0.87

The responsiveness of the MSQPT, i.e., its ability to measure change over time, has not yet been evaluated. Moreover, it is not yet clear how to interpret changes in MSQPT scores of the four items and sections of the MSQPT. This represents an important issue for using this tool in daily practice, as it is critical to estimate the minimal change that translates into real change (improvement or deterioration) in persons with MS.

The responsiveness (i.e., the ability to measure change over time) and interpretation of PRO measures have been topics of debate for many years. No established method exists to date, but there is a growing consensus regarding suitable approaches [5].

The anchor-based approach assigns patients to subgroups based on the degree of change (none, small, large); specifically, the change in the score given by the PRO instrument (PRO score) is compared with external evidence of change (real change), such as patient-based global rating of change questions. The change in PRO score for the patient subgroup reporting a small change represents an anchor-based estimate of the minimal important difference (MID) [6].

The distribution-based approach uses various statistical measures based on the distribution of the PRO scores in a given sample [7–9]. Distribution-based MID estimates can be calculated based on the standard deviation (SD) [5, 6, 10–12], standard error of measurement (SEM) [6, 13] or minimal detectable change (MDC) for the 90% (MDC_90_) and 95% (MDC_95_) confidence intervals [6]. When small effects are expected, the SEM is estimated as the standard deviation of PRO scores multiplied by the square root of the difference between one and the intraclass correlation reliability coefficient [13]. By combining the distributional and reliability components, SEM takes into consideration that some of the observed change may be caused by random measurement errors. Thus, the SEM measures response stability. Wyrwich et al. [13] stated that the one-SEM criterion can be applied to detect intra-individual change using health-related quality-of-life instruments.

The MDC represents another statistical estimate of the smallest change that can be detected by an instrument and is calculated as the product of SEM, the square root of 2 and 1.96 or 1.26 for MDC_95_ and MDC_90_, respectively. The MDC gives the smallest amount of change beyond random error for a certain level of confidence. It is always higher than SEM because it is calculated as SEM multiplied by the square root of 2 and 1.96 or 1.26.

The anchor-based and distribution-based MID estimates represent different concepts of establishing a value for minimal change. The anchor-based concept uses external clinical information from the patient or clinician to express minimal change. The distributional method relies solely on statistical calculations and does not directly inform about minimal clinical change. Both Revicki [5] and Turner [6] recommended the use of both concepts to establish an MID, giving more weight to the anchor-based MID estimate and using the distribution-based estimates as benchmarks. Turner et al. [6] showed that 0.5 and one SEM come closest to the anchor-based estimates and that the MDC cannot replace an anchor-based MID.

The effect size (ES) measures the change caused by an intervention as the difference between the mean scores obtained during the pre- and post-intervention assessments divided by the SD of the baseline scores. ES values of 0.2, 0.5 and 0.8 indicate small, medium and large changes, respectively. The standardized response mean (SRM) is considered a more informative measure than the ES, as it uses the SD of change in scores between assessments (instead of the SD at baseline) in the denominator, taking the variability of change into account. The modified SRM (MSRM) uses the same numerator as the ES and SRM, but the denominator is the SD of change in scores between assessments calculated only for those individuals who are identified as stable based on independent external information, typically provided in the form of a rating of change question during the post-intervention assessment. The MSRM provides us with an estimate of the inherent variability of changes recorded by the PRO instrument, with lower scores indicating lower variability [7].

Further information on the responsiveness of a PRO instrument can be obtained using the relative efficiency method, which compares the responsiveness of two PRO instruments. The relative efficiency is calculated as the square of the ratio of the t-statistics for the two instruments being compared, thus revealing which instrument is more responsive in a given survey population.

The purpose of this study was to evaluate the responsiveness of the German MSQPT and to establish reasonable estimates of MID in order to provide practical guidelines on how to interpret changes in MSQPT scores.

## Methods

We used a longitudinal multicentre design with a convenience sample. Eleven private practices and two physiotherapy departments of hospitals in Switzerland participated in this study. The physical therapists of each participant institution recruited the patients.

### Inclusion criteria

We included patients who were diagnosed with MS according to the McDonald criteria, undergoing physiotherapeutic treatment for MS, older than 18 years, able to read the MSQPT, native German speakers and given an Expanded Disability Status Scale (EDSS) score of ≤6.5.

### Exclusion criteria

Patients were excluded if they presented acute exacerbation of MS, any condition that made them bedridden, distinct fatigue that made it impossible to concentrate for ≥2 h or grave cognitive change (judged by the treating physical therapist).

The execution of the testing was standardized using a study manual with detailed instructions. The two testers were experienced in using the MSQPT and HAQUAMS, as they were testers in the validation study for the MSQPT [1]. They were familiarized with the study manual and were trained by experts in physical testing for evaluating the patients in a standardized manner, as instructed in the study manual.

### Outcome measures

The anchor-based approach usually measures change by employing one global rating of change question with a symmetrical scale of 7 to 15 points [14]. Depending on the width of this scale, a change of one or two points may represent a minimal change. However, anchor-based estimates are always flawed with the uncertainty of the value indicating global change (real change). The wide range of symptoms experienced by MS patients can make it especially difficult to assess the extent of change on one global rating scale [14]. For example, when asking whether the patient generally feels better or worse, we might not register an amelioration in walking if, at the same time, the pain worsened. One should always ask oneself whether a single question assessing the global change is sufficiently sensitive. We used several patient-based and therapist-based questions to rate the change in order to obtain a clearer view of real change in the patients. The questions assessing change described issues relevant to the physiotherapeutic treatment, such as pain, fatigue, walking and balance, as well as therapeutic goals, such as improved activity and participation, which lie at the core of MSQPT. By formulating the questions in this way, we ensure that we are comparing similar constructs (various items and sections of MSQPT vs. questions rating global change). Furthermore, a more detailed rating system, assessing various symptoms separately, may also serve to ensure that the comparison is relevant [5, 15–17]. However, this implies the use of a multiple rating approach, which results in a range of MID estimates [5, 14].

The detailed questions on global rating of change were provided on two different questionnaires considering two different perspectives: that of the patient and that of the treating therapist. These two questionnaires differed only regarding phrasing of the questions, not regarding the matter being asked. Each questionnaire had 9 rating of change questions that concerned general health status, balance, walking ability, arm function, fatigue, pain, activity level, social participation and general impairment due to MS. The first question for the patient was: “Compared to the situation before the first testing, how would you describe your general health now”? The other 8 questions were similarly phrased, varying the topic as listed above. Furthermore, each question had a 9-level scale, similar to the one in the MSQPT, with the extremes “much worse” and “much better”, and the middle level being “the same”.

The HAQUAMS is a reliable, valid and responsive instrument [18, 19]. It is a German self-rated quality of life questionnaire developed for use in an MS population. In the MSQPT validation study [1], the HAQUAMS showed good correlations with the main groups of the MSQPT.

The patients were evaluated using the following physical tests: the 6-Metre Timed-Walking Test (6MTWT), 6-Minute-Walk Test (6MWT) and Berg Balance Scale (BBS). The 6MTWT and 6MWT were standardized using a static start, and patients were asked to walk at a comfortable, usual speed [8, 20, 21] to ensure safety, as many tests were executed in the confined space of private practices. A 20% change was used as the threshold for change [21–24].

The BBS was standardized using a conservative protocol, in which the lowest of two levels was given in cases of uncertainty. Each test was demonstrated to the patients by the testers during its testing session, in agreement with the test manual. A 7-point change in BBS was the limit used for real change [25].

### Procedures

To compare the study population with those of former studies in Switzerland [1, 26] and assess the representativeness of the population, we recorded age, gender, type of MS and disease duration since diagnosis of the patients at baseline. Furthermore, patients were allocated to groups according to their score in the EDSS at baseline. The tests were executed in the following order: MSQPT, 6MTWT, BBS, HAQUAMS, patient rating of change questionnaire and 6MWT. Patients were allowed to have a break at any time, and the break times were recorded. The treating therapist filled out the rating of change questionnaire during or after the testing session, without any contact with the patient.

The usefulness of the MSQPT may be different for the different treatment situations in Switzerland. Both long-term and short-term treatments were included in order to evaluate whether the MSQPT is useful for all treatment situations. I considered long-term patients to be those who were in physiotherapeutic treatment for one year or more. These patients may show little change over time, and advancement of quality of life is central for treatment. The long-term patients were tested twice, once at baseline and once 6 months later. Short-term patients were considered those who underwent 9–27 treatment sessions. These patients were tested at baseline and after 3–4 months or at the end of the treatment period if the latter period was shorter.

All patients were in a non-standardized physiotherapeutic treatment. The treatment was individually tailored depending on the presented symptoms, and the goals for each therapy were determined by the patient and therapist together.

### Data analysis

The full dataset was subjected to analysis. No subgroup analyses for short- and long-term patients were executed for this analysis. The responsiveness of the MSQPT was assessed using ES and SRM for patients with change and using MSRM for patients without change in the results. I computed the relative efficiency between the MSQPT and the HAQUAMS scores for amelioration and deterioration. The t-statistics of the items and sections of the MSQPT were used in the numerator, and the t-statistics of the groups of the HAQUAMS were used in the denominator. A relative efficiency of >1 indicated that the MSQPT was more responsive than the HAQUAMS, whereas a relative efficiency of <1 indicated the opposite.

A combined distribution- and anchor-based approach was used to establish an MID. For the distribution-based MID estimates, 0.2*SD, 0.33*SD, SEM and the MDC were calculated for the 90% and 95% confidence intervals (MDC_90_ and MDC_95_, respectively). Although 0.5*SD is the best choice for SD [6, 11, 12], it is equivalent to 1 SEM [12] or greater [6] and therefore does not contribute additional information. Thus, 0.2*SD was chosen, as is often used [5, 10], and 0.33*SD was used for comparable reasons, as the MID of the HAQUAMS was based on 0.33*SD [11]. They represent the lowest distribution-based MIDs in this study.

For anchor-based estimates, it is important that there be a reasonable correlation between baseline and final testing [5, 27, 28]. I calculated the correlation coefficients to assess whether this requirement was fulfilled. The anchor-based values were considered reasonably correlated and were used in the evaluation when the coefficient was ≥0.30 [1]. Furthermore, in this analysis, I considered only global rating of change questions for items, sections or total scores of the MSQPT that had a similar content to the global rating of change questions. Table 3 shows the linking between items, sections and total score and the global rating of change questions with similar content. This linking will be used for MSRM (using anchor-based information for no change) and anchor-based MID estimates.

**Table 3:** Linking of items, sections and total score to global rating of change questions

Global rating of change questions	Items, section and total score of MSQPT							
	M4	M8a	M8b	M10	Activity	Participation	Balance	Total score
Global health					*	*		*
Balance	*	*	*	*			*	
Walking	*	*	*	*				
Participation					*	*		*
Impairment					*	*		*

*M4* taking a shower, *M8a* walking distance, *M8b* walking time, *M10* taking a shower, * displaying similar content

The anchor-based MIDs for the 4 items, 3 sections and the total score were determined using the global ratings of change in the rating of change questionnaires. Changes of one or two levels in the global rating of change questions were classified as minimal differences. For all patients exhibiting a one- or two-level change, the average change of the items and sections of the MSQPT between baseline and final testing was computed.

The anchor- and distribution-based MIDs present a range of MID estimates for each item, each section and the total score. I expressed all distribution- and anchor-based estimates of MID in integer numbers, rounding the MID to the next higher number, because the answer scales of the MSQPT correspond to integer numbers.

To narrow the range of all calculated MIDs to possibly one value, the sensitivity and specificity statistics of all the MID scores were used.

The sensitivity described the agreement regarding change for each item and section between the assessments and the true change rate given by the global rating of change. The specificity described the agreement regarding no change for each item and section between the assessments and the absence of change as measured by the global rating of change. Thus, the sensitivity and specificity for amelioration and deterioration were calculated for each MID of the items, sections and total score based on the global ratings of change given by the patients and therapists.

The best MID was defined as the MID with the best values of sensitivity and specificity. The best MID for an item of MSQPT was obtained by choosing the MID out of the whole range of integer MIDs of that item that had the highest sensitivity and specificity values for the global ratings of change that had similar content. If two values had a similar range of sensitivity and specificity, the lower value was chosen as the best MID. In addition, the sensitivity and specificity based on the patients’ rating of change questions were given more weight than those based on the therapist rating. The same method was used for the sections and total score.

Finally, the best MIDs for the 4 items of Table 1 and for the balance section were compared with real changes as seen in physical tests.

In the discussion, I examine the method and the value of the findings.

When benchmarking the best MID with anchor- and distribution-based values, I follow the recommendations [5, 6], giving the anchor-based approach more weight. It will be assumed that the anchor-based estimate will be of similar value to the SEM [6, 13]. As the 0.5 SD might be similar to SEM [6, 12], the 0.2 and 0.33 SD might be lower, and the MDC will be higher than SEM. In addition, other characteristics can be taken into account, such as the distribution of scores (floor and ceiling effects) and reliability [7]. If an item has a ceiling effect, it is hard to show improvement. Furthermore, a low MID is more plausible for a very highly reliable item or section than when the reliability is low.

## Results

### Demographic data

Sixty-one patients from thirteen test locations were included in the study. All patients provided informed consent. However, due to a new and serious diagnosis, one patient in long-term treatment decided not to continue in the study.

Of the 60 remaining patients, 25 in long-term treatment and 35 in short-term treatment finished the study. Moreover, 53 patients were treated in private practice, while 7 were treated in a hospital setting.

The population of the study has almost the same percentage of women and range of age as the validation study of the MSQPT [1] and as the Multiple Sclerosis and Rehabilitation, Care- and Health Services study (MARCH), the Swiss contribution to the international research programme to close gaps in the knowledge of the living conditions of persons with MS [26] (Table 4). The main difference is that in this study, the average age, the mean age of male patients and the percentage of patients over 60 years old were slightly higher, while the percentage of patients between 40 and 60 years old was slightly lower. I concluded that the sample has a population comparable to the validation study of the MSQPT and to the MARCH study of Switzerland and is plausibly representative of the Swiss MS population.

**Table 4:** Demographic data of the MARCH study, the validation study of the MSQPT and the current study

Demographic data	MARCH study Switzerland all data	MARCH study Switzerland physical therapy	Validation study MSQPT	Responsiveness and MID study MSQPT
Percent women	63	58	63	65
Mean age	50.2 (±11.9)	51.2 (±11.4)	51.7 (±10.4)	53.3 (±11.4)
Mean age women	49.8	52	50.8	52.6 (±12.5)
Mean age men	50.1	49.5	53.3	54.5 (±9.0)
Min-max age	16–79	26–79	29–84	23–77
Percent 40–60 years	60	60	62	53
Percent ≥ 60 years	22	21	24	30
N	185	113	141	60
Mean years of illness	13	Not available	15	18 (±9.7)

### Percentage of missing data

The MSQPT and HAQUAMS had very low rates of missing data (0.13 and 0.47%, respectively). Moreover, the patient rating of change question in the MSQPT and HAQUAMS did not exhibit missing data. The therapist rating of change questionnaire had a 5.4% missing data rate. The patient rating of change questionnaire was completed by 77% of the patients. Statistics were calculated excluding patients with missing data from the corresponding dataset.

### Evaluation of responsiveness

The distribution-based estimates of the ES and SRM are shown in Table 5 for data with negative change items (deterioration) and positive change items (improvement) between baseline and final testing scores. The ES showed low deterioration for the activity section items (−0.46), medium for the M4 item (getting in and out of a car, −0.67), the participation section items (−0.64) and total score (−0.58) and high deterioration for the other items. Regarding improvement, except for item M10, which exhibited a high ES (1.49), most ES values were similar to the deterioration items. Each SRM was higher than its corresponding ES except for M4.

**Table 5:** Responsiveness of questionnaire items and sections with respect to physiotherapy-related deterioration and improvement in patients

		M4	M8a	M8b	M10	Activity	Participation	Balance	Total Score
Deterioration	n	3	19	19	6	32	28	18	29
	ES	−2.84	−0.91	−1.03	−0.67	−0.46	−0.64	−1.00	−0.58
	SRM	−1.23	−3.09	−2.57	−1.12	−0.82	−1.21	−1.27	−0.96
Improvement	n	1	16	17	5	22	28	19	31
	ES	*	1.02	0.80	1.49	0.49	0.75	1.04	0.57
	SRM	*	1.92	2.20	1.41	1.38	1.48	1.68	1.29

*ES* effect size, *SRM* standardized response mean, *M4* showering, *M8a* walking distance, *M8b* walking time, *M10* getting in and out of the car

*Not computable (*n* = 1)

The group of people without change, as identified using the global rating of change questions, was used for the evaluation of MSRM, which should be as low as possible. Table 6 shows the MSRM for each item of Table 1, each section and the total score (rows) based on the different global ratings of change for both patient and therapist answers (lines). The number of patients who were without change was different for each global rating of change question. The MSRMs were generally low except for Item M4. The activity section exhibited the highest MSRM among the sections.

**Table 6:** Modified standardized response mean for multiple sclerosis patients identified as stable based on questions assessing the global rating of change

Item, section and total score of the MSQPT	M4	M8a	M8b	M10	Activity	Participation	Balance	Total score
Global rating of change questions								
Perspective of the patient								
Global change	*	*	*	*	0.15	0.00	*	0.03
Balance	0.27	−0.04	0.12	0.10	*	*	0.03	*
Walking	**	−0.04	0.17	0.10	*	*	*	*
Participation	*	*	*	*	0.12	0.00	*	0.03
Disability	*	*	*	*	0.14	0.00	*	0.03
Perspective of the therapist								
Global change	*	*	*	*	0.12	0.00	*	0.03
Balance	0.60	−0.05	0.13	0.06	*	*	0.04	*
Walking	0.26	−0.05	0.11	0.07	*	*	*	*
Participation	*	*	*	*	0.12	0.00	*	0.03
Disability	*	*	*	*	0.14	0.00	*	0.03

*M4* taking a shower, *M8a* walking distance, *M8b* walking time, *M10* getting in and out of the car, * not applicable, ** not computable

Tables 7 and 8 show the relative efficiency between the MSQPT and HAQUAMS scores. The data of Table 7 show that the MSQPT total score seems to be as responsive as the HAQUAMS total score for showing improvement. The participation section of the MSQPT was better at indicating improvement than each HAQUAMS section. In contrast, the activity section of the MSQPT was much less efficient in showing improvement than the corresponding HAQUAMS sections. When improvement in walking was compared, item M8a of the MSQPT (“How far can you walk without a rest”?) was more responsive than the mobility factors of the lower limb in the HAQUAMS, while in the same comparison, the M8b of the MSQPT (walking time) was less responsive.

**Table 7:** Relative efficiency between the MSQPT and the HAQUAMS with respect to improvement

	MSQPT				
HAQUAMS	M8a Walking distance	M8b Walking time	Activity	Participation	Total score MSQPT
Fatigue/thinking	*	*	*	1.28	*
Lower limb mobility	1.48	0.84	0.37	1.07	0.70
Upper limb mobility	*	*	0.36	1.05	0.68
Social functioning	*	*	*	1.35	*
Mood	*	*	0.51	1.47	0.96
Total score HAQUAMS	*	*	0.52	1.51	0.98

*MSQPT* Multiple Sclerosis Questionnaire for Physiotherapists, *HAQUAMS* Hamburg Quality of Life Questionnaire in Multiple Sclerosis. Only the combinations for which the correlation is >0.4 are shown. *These sections do not assess similar aspects

**Table 8:** Relative efficiency between the MSQPT and the HAQUAMS with respect to deterioration

	MSQPT				
HAQUAMS	M8a Walking distance	M8b Walking time	Activity	Participation	Total score MSQPT
Fatigue/thinking	*	*	*	2.24	*
Lower limb mobility	1.48	3.83	0.92	2.46	1.75
Upper limb mobility	*	*	0.95	2.53	1.80
Social functioning	*	*	*	1.84	*
Mood	*	*	0.69	1.85	1.32
Total score HAQUAMS	*	*	0.66	1.76	1.25

*MSQPT* Multiple Sclerosis Questionnaire for Physiotherapists, *HAQUAMS* Hamburg Quality of Life Questionnaire in Multiple Sclerosis. Only the combinations for which the correlation is >0.4 are shown. *These sections do not assess similar aspects

The MSQPT total score was more effective in demonstrating deterioration than the HAQUAMS total score (Table 8). Furthermore, regarding deterioration, the MSQPT participation section was clearly more responsive than any HAQUAMS score. The MSQPT activity section showed deterioration with similar efficiency to that of the mobility sections but less efficient than the total score of the HAQUAMS. Both mobility items of the MSQPT were more responsive than the lower limb mobility section of the HAQUAMS.

### Estimates for MID

Table 9 shows the distribution-based estimates for MID. There was a considerable difference regarding the use of SD, SEM or MDC statistics, with the first two resulting in much lower values than those obtained using the MDC. Regarding the MSQPT, the distribution-based MID estimates of item M4 were 1 or 2. The other MID estimates were between 1 and 3 for items M8a, M8b and M10; between 4 and 19 for the activity section; between 3 and 22 for the participation section; between 1 and 5 for the balance section; and between 7 and 45 for the total score.

**Table 9:** Distribution-based estimates of the minimal important difference

	M4	M8a	M8b	M10	Activity	Participation	Balance	Total score
0.2*SD	0.25	0.30	0.33	0.18	3.31	2.68	0.52	6.20
0.33*SD	0.41	0.51	0.56	0.30	5.52	4.46	0.86	10.33
SEM	0.67	0.81	0.85	0.77	6.64	7.66	1.70	15.93
MDC_95_	1.87	2.24	2.39	2.13	18.40	21.23	4.70	44.16
MDC_90_	1.57	1.89	1.98	1.80	15.49	17.87	3.96	37.17

*SD* standard deviation, *SEM* standard error of measurement, *MDC*_*90*_ minimal detectable change of the 90% confidence interval, *MDC*_*95*_ minimal detectable change of the 95% confidence interval, *M4* taking a shower, *M8a* walking distance, *M8b* walking time, *M10* getting in and out of the car

Before calculating the anchor-based MID estimates, I tested whether the items and sections exhibited a minimum and substantial correlation between baseline and target score. All correlation coefficients except that for item M10 fulfilled the minimum requirement of *r* = 0.30, with r of 0.57–0.84, *p* < 0.0001. For this reason, item M10 was excluded from the anchor-based MID calculations.

Table 10 shows the anchor-based MID. These results represent the average change between baseline and final testing, using the MSQPT sections and items, for MS patients with deterioration or improvement that were identified by the rating of change questionnaires as having a change level of 1 or 2. The table shows the pairs of items and sections that seemed to exhibit a meaningful relationship as described in Table 3.

**Table 10:** Anchor-based estimates of the minimal important difference predicting change with respect to deterioration and improvement

Global rating of change questions	Item/Section of the MSQPT						
	M4	M8a	M8b	Activity	Participation	Balance	Total score
Therapist, global rating, deterioration (35)	*	*	*	−3.09	−3.40	*	−9.09
Therapist global rating, improvement (18)	*	*	*	3.00	2.00	*	7.78
Patient, global rating, deterioration (41)	*	*	*	−2.17	−1.71	*	−5.02
Patient, global rating, improvement (12)	*	*	*	3.63	1.17	*	4.96
Therapist, walking rating, deterioration (41)		−0.46	−0.37	*	*	*	*
Therapist, walking rating, improvement (12)		0.58	0.08	*	*	*	*
Patient, walking rating, deterioration (15)		−0.13	−0.53	*	*	*	*
Patient, walking rating, improvement (6)		0.67	0.67	*	*	*	*
Therapist, balance rating, deterioration (30)	−0.27	−0.50	−0.47	*	*	−1.00	*
Therapist, balance rating, improvement (18)	0.22	0.28	0.06	*	*	0.17	*
Patient, balance rating, deterioration (9)	−0.11	−0.22	−0.67	*	*	−1.78	*
Patient, balance rating, improvement	**	**	**	**	**	**	**
Patient, participation rating, deterioration (8)	*	*	*	−1.71	4.86	*	−6.86
Patient, participation rating, improvement (6)	*	*	*	4.00	2.00	*	14.57
Therapist participation rating, deterioration (7)	*	*	*	−7.63	−11.00	*	−21.88
Therapist participation rating, improvement (7)	*	*	*	5.67	5.00	*	16.50
Patient, impairment rating, deterioration (12)	*	*	*	−12.20	−2.80	*	−24.40
Patient, impairment rating, improvement (9)	*	*	*	2.00	−3.00	*	2.20
Therapist, impairment rating, deterioration (5)	*	*	*	−6.08	−1.42	*	−10.42
Therapist, impairment rating, improvement (5)	*	*	*	2.22	3.33	*	2.33

*M4* taking a shower, *M8a* walking distance, *M8b* walking time; () displays the number of cases; *rating and section are not relevantly related; **not computable

The MID estimates of the activity section were between 2 and 13, those of the participation section were between 2 and 11, those of the activity section were between 1 and 2 and those of the total score were between 2 and 25. The estimates for the items were all well below 1, the lowest possible score for the MSQPT items.

Sensitivity and specificity were assessed for the whole range of MID described in Tables 9 and 10. All MIDs were rounded to the next integer number. Table 11 shows the MIDs for deterioration and amelioration for the items. The best MID (in bold), presenting the best sensitivity and specificity, and the sensitivity and specificity for the next level of the item are shown. As outlined in the method section, if 2 levels showed similar values, the lower MID was chosen as the best MID. Those sensitivity and specificity values were used and shown if the data for at least 4 persons were available. Values were used in the description of the results if and only if an item (or section) had a similar content to a rating of change question (see Tables 9 and 10, column 2). Both the perspective of the patient and the perspective of the therapist were used.

**Table 11:** Sensitivity and specificity of the estimates for minimal important difference (MID) for 4 items

Item	Global rating questions used	MID improvement	Specificity	Sensitivity	MID deterioration	Specificity	Sensitivity
M4	Balance, Arm function	*1*	0.52–0.81	*	*1*	0.52–0.81	0.20–0.67
		2	*	*	2	*	*
M8a	Walking, Balance	*1*	0.40–0.71	0.1–0.25	*1*	0.45–0.71	0.36–0.67
		2	0.47–0.69	0.1–0.3	2	0.40–0.68	*
M8b	Walking, Balance	*1*	0.47–0.65	**	*1*	0.47–0.65	0.32–0.53
		2	0.42–0.70	0.0–0.5	2	0.43–0.70	0.36–0.83
M10	Walking, Balance	1	0.41–0.74	0.00–0.33	1	0.41–0.74	0.36–0.60
		*2*	0.43–0.74	*	*2*	0.4–0.80	0.67–0.8
		3	*	*	3	*	*

*M4* taking a shower, *M8A* walking distance, *M8B* walking time, *M10* getting in and out of the car; *n<4; **not computable, *** no values available. Italic numbers represent the best MID

The integer MID for item M4 ranged from 1 to 2. There were not enough data (*n* < 4) for calculating more than one level change in the item. The best MID for improvement and for deterioration of item M4 was set at one.

The Walking items had an integer MID range of 1–3. The items had a similar level for the sensitivity and specificity of the one- or two-level change in the item. Only the sensitivity for a two-level deterioration for item 8b showed one clear higher value (0.83) than a one-level change. The best MID was set at one level of change.

Only distribution-based MID estimates were available for item 10, with an integer range from 1 to 3. A three-level change could not be calculated for item M10 (n < 4). Because the sensitivity for deterioration with a 2-level MID was generally higher than for level one, the MID was set at a two-level change.

Table 12 shows (similar to Table 11) sensitivity and specificity values of the best MIDs of the sections and total score and the adjacent available values.

**Table 12:** Sensitivity and specificity of estimates for minimal important difference (MID) for the sections and total score of the MSQPT

Section	Global rating questions used	MID improvement	Specificity	Sensitivity	MID deterioration	Specificity	Sensitivity
Activity	Global health, Impairment, Walking	6	0.25–0.65	0.10–0.67	10	0.36–0.72	0.39–0.70
		*7*	0.29–0.86	0.12–0.75	*11*	0.44–0.78	0.33–0.78
		8	0.28–0.86	0.13–0.75	25	0.42–0.72	*
Participation	Global health, Impairment, Participation	16	0.47–0.90	0.50	15	0.44–0.9	0.22–0.78
		*17*	0.45–0.88	0.67	*17*	0.43–0.91	0.40–0.80
		20	0.45–0.89	0.50	22	*	*
Balance	Balance	1	0.46, 0.69	0.13, 0.15	2	0.42, 0.69	0.42, 0.42
		*2*	0.48, 0.68	0.10, 0.21	*3*	0.49, 0.72	0.5, 0.67
		3	0.49–0.71	0.13, 0.25	4	*	*
Total score	Global health, Participation Impairment	16	0.44–0.97	0.11–0.66	16	0.26–0.65	0.17–0.68
		*20*	0.47–0.95	0.14–0.80	*18*	0.29–0.65	0.21–0.67
		25	0.47–0.95	0.20–0.80	22	0.29–0.64	0.25–0.75

*n<4; **not computable. Italic numbers represent the best MID

The activity section displays integer anchor- and distribution-based MID estimates ranging from 3 to 19. Of the available values for improvement, 7 levels of change provide the best MID, with higher sensitivity and specificity than 6 levels and similar sensitivity and specificity. The best MID for improvement was set at 7 levels. Because the sensitivity values for 11 were generally higher than for a 10-level change, the best MID for deterioration was set at 11 levels.

The participation section, with an integer range of MID estimates between 2 and 22, had a best MID of 17 for both deterioration and amelioration based on the sensitivity levels. The sensitivity for amelioration was based on only the therapist perspective for global change because other ratings had n < 4. For deterioration, a 17-level change generally represents more high values than a 15-level change.

The range of 1–5 levels of change for the MID of the balance section is a wide range for a section that consists of two items. There was a clear choice for a three-level best MID for deterioration based on the sensitivity results. The choice of a best MID for amelioration was difficult to make as all three levels were similar. The best MID level was set at two and will be discussed later.

The total score range for MID was 3–45. The sensitivity for improvement was the basis for the choice of a best MID of 20. The values of the MID for deterioration did not show a clear picture. Sensitivity for global health was highest (0.78) for an MID of 16 from the patient perspective but highest (0.75) for 22 from the therapist perspective. To select a best MID of 16 would contradict the higher values for participation and impairment from the therapist view for 22. A best MID of 18 for deterioration was the most balanced choice.

The best MIDs of the items and of the balance section were compared with the results of the physical tests. A 20% change was used as a threshold for change for the 6MTWT and 6MWT, and a 7-point change was used for real change in the BBS.

Only the specificity for the best MID was calculated against the real change of the physical tests because few patients exhibited change in the physical tests. The results are shown in Table 13. The MID values of the items showed a high specificity for the physical tests BBS and 6MWT and slightly lower specificity for the 6MTWT. The balance section showed a high specificity for the BBS and a clearly lower specificity for the walking tests.

**Table 13:** Specificity of the best MID of relevant items in relation to the physical tests

	6MTWT	BBS	6MWT
M4 (*1)	0.79 (7.6, 3.6, 4.2–26.2)	0.92 (49, 5.1, 33–56)	0.81 (238, 75.0, 65–462)
M8a (*1)	0.82 (8.0, 3.7, 4.6–22.4)	0.94 (49, 4.3, 38–56)	0.92 (228, 89.2, 96–462)
M8b (*1)	0.83 (7.1, 3.3, 4.0–22.4)	0.98 (50, 5.2, 33–56)	0.90 (254, 78.9, 114–462)
M10 (*2)	0.66 (8.4, 5.5, 3.0–46.0)	0.98 (48, 6.1, 25–56)	0.82 (223, 88.0, 34–462)
Balance (*2,3)	0.66 (7.5, 3.8, 4.2–24.8)	0.95 (49, 4.8, 33–56)	0.64 (229, 77.3, 60–462)

*6MTWT* 6-metre timed walking test (mean, SD and range in seconds), *BBS* Berg Balance Scale (mean, SD and range in points), *6MWT* 6-minute walking test (mean, SD and range in metre), *M4* taking a shower, *M8a* walking distance, *M8b* walking time, *M10* getting in and out of the car; * the best thresholds for the minimal important difference

## Discussion

The main finding of the present study is that the MSQPT is a responsive questionnaire. The proposed PRO score thresholds associated with minimal change are low, indicating the high responsiveness of the MSQPT.

Based on the ES and SRM, the MSQPT can measure change. Moreover, when MS patients did not experience change (as determined by questions assessing the global rating of change), the MSQPT hardly showed any change, and the MSRM values were low.

The HAQUAMS is a reliable, valid and responsive instrument [11, 18] that showed good overlap with the main sections of the MSQPT [1]. The relative efficiency of the MSQPT over the HAQUAMS in this cohort relied on the improved ability of the MSQPT participation section to detect change; however, the HAQUAMS proved better than the MSQPT activity section at detecting improvement. The total score of the MSQPT seems to be more suitable than that of the HAQUAMS for detecting deterioration. Similarly, the MSQPT items related to walking were more responsive than the HAQUAMS items on mobility of the lower limb. When comparing the responsiveness between measures, one must take into account that although the sections are related, they do not assess exactly the same phenomena. In this context, the MSQPT can measure change and does so as efficiently as the HAQUAMS.

The MDC estimates for MID were generally higher than the other estimates because MDC is based on SD and SEM but also considers the confidence interval. The SD and SEM were more similar to the anchor-based estimates, with MID for all studied items being lower than 1, which is the lowest possible integer MID. The MDC values were much higher, as was expected above.

The best MID was identified based on the sensitivity and specificity of the various MID estimates found in this study. Most values for specificity were high, and not all values for sensitivity could be calculated. Considering that the physical test suggested little change, we may conclude that this population was rather stable.

Furthermore, there was a clear difference between the perspective of the therapists and that of the patients, which indicates that using only therapist-based global ratings may lead to different conclusions than using patient-based global ratings. Future research should thus consider the choice of anchor.

The best MID estimates were all well lower than the highest MDC estimates but higher than most anchor-based estimates. In this study population, the procedure for establishing a clinically relevant MID seemed to offer a best MID close to anchor-based MIDs that rely on external evidence of change and that lie within the upper and lower limits set by the distribution-based approach, which relies solely on statistics of the distribution of changes in PRO scores. The value and credibility of the best MID are further discussed in detail, weighing the existing evidence regarding the value of the items and sections included in the questionnaire and the results of the present survey, to reach a comprehensive conclusion.

Unlike the anchor-based estimates, distribution-based estimates provide a simple way of expressing change in a standardized statistic; however, such metrics are criticized as being only theoretical indicators, with no physical meaning [5]. Thus, MIDs estimated using only distribution-based metrics may not indicate a clinically meaningful minimal change. Combining both approaches may give an extensive overview of the ability of a PRO measure to detect change, but it also results in a wide range of possible MID estimates. When choosing a suitable MID out of a range of estimates to be used as a clinical threshold indicating change, the highest weight was given to the anchor-based estimates assessed from the patient’s perspective [5, 6].

The database for estimating the anchor-based MID derived from M4 (ability to shower independently) is small. First, many patients rated the maximum score, which indicates that the patient rating for minimal balance improvement (anchor estimate) was absent. For the same reason, the ES for improvement could not be calculated. The ES for deterioration was high (though based on only *n* = 3 measurements), indicating a high responsiveness. The few anchor-based MIDs (for deterioration and amelioration) as well as the SDs and SEMs were well below one. Considering that SEM may be close to the anchor-based estimate [7, 8], it seems appropriate to take a 1-point change as the estimate of MID. The very high reliability of this item supports this choice. The 1-point MID for deterioration had a sensitivity of 0.6 and a specificity of 0.52 based on the balance-related global rating (which is relevant to M4) and 0.92 based on the BBS. However, because the high MSRM that did not indicate a small MID and the database for this 1-point MID was small, further research should clarify the MID threshold for this item.

The items concerning walking (M8a and M8b) had similar content and showed similar results. They had high responsiveness, with high ES for deterioration (−0.91 and −1.03, respectively) and improvement (1.02 and 0.8, respectively), and low MSRM for MS patients who did not show change following physiotherapy (0.03–0.05 and 0.11–0.17, respectively). The anchor-based MIDs for walking were also quite low and under 1. The fact that the SDs and SEMs were low and that these items were also highly reliable [1] and more suitable for detecting change compared to the HAQUAMS questions regarding lower limb mobility, suggests that it is appropriate to consider a 1-point change as the clinically relevant MID threshold. Only the MDCs (1.89–2.39) suggest that the MID might be higher.

Regarding the walking-related item M8a and the patient perspective for Walking, there was a sensitivity of 0.20 for improvement and a better sensitivity of 0.5 (M8b: 0.53) for deterioration. The specificity values were 0.50–0.53 for both M8a and M8b. A higher specificity (0.82–0.98) was found with respect to the physical tests (BBS, TWT and 6MWT).

An anchor-based estimate was not calculated for item 10. The given MID was mainly computed from the distribution-based statistics. The medium ES for deterioration (−0.67), low MSRM (0.04–0.13) and low MIDs (only the MDC_95_ > 2) suggests that a 2-point change is a reasonable threshold for a clinically relevant MID.

The activity section provided moderate ES (−0.46 and 0.49 for deterioration and improvement, respectively) and low MSRM (0.12–0.15), showing that this section is responsive. The best MIDs were 11 for deterioration and 7 for improvement. The SD and SEM estimates were lower than 7, and the integer MDC values were 16 and 19. The anchor-based MID estimates for deterioration were 3.09 to 6.08 (therapist-based) and 2.17–12.2 (patient-based). The best MID of 11 for deterioration was close to the upper limit of the anchor- and patient-based MID, higher than SEM and lower than the MDC. Weighting anchor-based MID estimates more heavily, 11 was a more conservative value. It had a sensitivity of 0.50 (specificity of 0.48), calculated from the patient-based ratings of global health, and a specificity of 0.7–0.8 for impairment.

The anchor-based estimates for improvement were 2.22–5.67 (therapist-based) and 2.00–4.00 (patient-based). A best MID of 7, equal to the SEM, was over the upper limit of all anchor-based MID estimates but much lower than the MDC values. The best MID of 7 was more reflective of an anchor-based estimate and was a reasonable, but considering MDC, also an optimistic choice, which requires further evaluation. It had a sensitivity of 0.75 (specificity of 0.57) calculated from the patient-based ratings of global health and a specificity of 0.70–0.89 for impairment.

The participation section also showed high responsiveness, with moderate ES (−0.64 and 0.75 for deterioration and improvement, respectively) and very low MSRM (0.00). The best MID was 17 for both deterioration and improvement. The distribution-based benchmarks SD and SEM were below 9, and the MDCs were 18 and 22. The anchor-based estimates for deterioration were quite low, ranging from 1.42 to 11, and those for amelioration ranged from 1.17 to 5. Based on anchor-based MID and SEM, a lower best MID than 17 could be expected. However, it is important to note that the patient rating for worsening participation was positive, while the patient rating for improvement of impairment was negative. This might be explained by the fact that the participation section had items that were indirectly related to participation and therefore was not identical to participation as rated by the patients. Change in these items may have caused this phenomenon. These two values should be viewed with caution. Furthermore, the sensitivity was based on few values that were mainly therapist-based. Additionally, the participation section had a reliability of 0.77 [1] that did not speak in favour of a low MID. Because of these reasons, an MID of 17 seems to be a reasonable estimate for the clinically relevant threshold. The sensitivity for deterioration was 0.8 for participation rating from the perspective of the therapist, while its specificity for participation was 0.91 (therapist) and 0.76 (patient). The sensitivity for amelioration was based on only the global change of health question (0.8, therapist perspective). Its specificities for participation were 0.88 (therapist) and 0.65 (patient).

The balance section showed high responsiveness, with high ES (−1.00 and 1.04 for deterioration and improvement, respectively) and very low MSRM (0.03 and 0.04). The best MIDs for improvement (2 points) and deterioration (3 points) were considerably higher than the anchor-based estimates (−1.78 and 0.17, respectively) and SEM (1.70) but lower than the MDCs (3.96 and 4.70, respectively). Taking into account that these MIDs have high specificity with respect to the BBS (0.95) and sufficient test-retest reliability (0.84) [1], these MID thresholds are well justified.

The total score of the MSQPT was responsive to change, with medium ES (−0.58 and 0.57 for deterioration and improvement, respectively) and very low MSRM (0.03). The best MID for deterioration (improvement) was 18 (resp. 20). The distribution-based benchmarks were 6.20 and 10.33 for SD, 15.93 for SEM and 37.17 and 44.16 for the MDCs. The anchor-based MIDs for deterioration ranged from 5.2 (patient perspective, global rating) to 24.4 (patient perspective, impairment rating). This MID was higher than SEM, SD and the anchor-based MID except for impairment. Because the total score had a reliability of 0.87 [9], this low best MID appears to be an anchor-based, reasonable but optimistic estimate for the clinically relevant threshold. It had a sensitivity of 0.67 and a specificity of 0.3 for global health (both patient perspective). The anchor-based MIDs for amelioration ranged from 2.20 to 16.60. The upper limit was similar to SEM, higher than SD and much lower than the MDC. The MID also seems to be an anchor-based, reasonable and optimistic MID. It had a sensitivity of 0.60 and a specificity of 0.47 for global health (both patient perspective).

These proposed MIDs had very different absolute sizes. If we set them in relation to the maximum value of each item and section for which the MID was proposed and calculate the percentage of change, we can better appreciate their value. Table 14 shows these percentages of change.

**Table 14:** Best MID in percentage of maximum value

	Improvement		Deterioration	
Item/Section	Best MID	Best MID in %	Best MID	Best MID in %
M4	1	11%	1	11%
M8a and M8b	1	10%	1	10%
M10	2	22%	2	22%
Activity	7	5.4%	11	8.6%
Participation	17	17.2%	17	17.2%
Balance	2	11%	3	16.6%
Total score	20	6.3%	18	5.7%

*M4* taking a shower, *M8a* walking distance, *M8b* walking time, *M10* getting in and out of the car

The proposed MIDs could detect from 5.4% (activity section) to 22% change with respect to improvement and from 5.7% (total score) to 22% change with respect to deterioration. These MIDs are low, and most of them are more anchor- than distribution based.

The MSQPT was validated against the SF-36 and the HAQUAMS, another German PRO instrument specially tailored for MS patients. The MSQPT performed similarly to the HAQUAMS against the SF-36 [1]. In the present study, the MSQPT showed comparable responsiveness (relative efficiency) in relation to the HAQUAMS. MSQPT may detect small changes based on a 9- and 10-point answer scale, which is very important for the evaluation of the effect of physiotherapeutic treatment. These psychometric qualities make the MSQPT a very promising PRO instrument for the evaluation of outcomes of physiotherapy in MS patients.

This study uses a mixed population of which all persons were in short- or long-term treatment, and no predefined intervention was used. The focus of this study was the evaluation of responsiveness in a broad spectrum of therapy because the MSQPT is used in this way. The population of this sample was rather stable. To further evaluate the value of the proposed MID, a comparison of persons without treatment versus persons in treatment may bring important insights into the performance of the MID, especially if the population with treatment shows considerable change.

### Study limitations

The present study is limited in the following aspects. The study population, although representative, was relatively small and rather stable. It is not clear how the proposed MIDs will fare in a population exhibiting a higher degree of change and especially higher improvement. Some sensitivity values could not be calculated or were based on small numbers. The approach of using rating of change questions for the global ratings gave a range of sensitivity and specificity values that were not fully coherent; thus, the choice of the best MIDs based on a range of sensitivity and specificity values was partly arbitrary. Only 77% of the patients filled out the questionnaire regarding global rating of change.

## Conclusions

The present study showed that the MSQPT is responsive and can detect physiotherapy-induced changes in MS patients. The proposed MIDs are reasonable estimates that may be used in daily practice as clinical thresholds indicating change. Further research in an MS population exhibiting considerable change is needed to provide more data to understand how the proposed thresholds perform in detecting change.

## Additional files

Additional file 1: Shows the most up-to-date German MSQPT. (PDF 435 kb)
Additional file 2: Shows the English translation the items of the MSQPT. (PDF 98 kb)

## Abbreviations

6MTWT: 6-metre timed walking test
6MWT: 6-minute walk test
BBS: Berg balance scale
EDSS: Expanded disability status scale
ES: Effect size
HAQUAMS: Hamburg quality of life questionnaire for multiple sclerosis
MARCH: Multiple sclerosis and rehabilitation, care- and health services
MDC: Minimal detectable change
MDC_90_: Minimal detectable change for the 90% confidence interval
MDC_95_: Minimal detectable change for the 90% confidence interval
MID: Minimal important difference
MS: Multiple sclerosis
MSIS-29: Multiple sclerosis impact scale
MSQPT: Multiple sclerosis questionnaire for physical therapists
MSRM: Modified standardized response mean
PRO: Patient rated outcome
SD: Standard deviation
SEM: Standard error of measurement
SF-36: Short form health survey
SRM: Standardized response mean
