# Supplementary material for: Patient-reported questionnaires in MS rehabilitation: responsiveness and minimal important difference of the multiple sclerosis questionnaire for physiotherapists (MSQPT)
Source: BMC Neurol. 2017 Mar 16;17:50. doi: 10.1186/s12883-017-0834-1 (PMC5356359; doi:10.1186/s12883-017-0834-1)
Supplement: Additional file 1: — Shows the most up-to-date German MSQPT. (PDF 435 kb) [file 12883_2017_834_MOESM1_ESM.pdf]

MSQPT ®

Multiple Sclerosis Questionnaire for Physiotherapists Patienten-Fragebogen zur Therapieevaluation

Wir möchten Ihnen die bestmögliche Therapie anbieten. Zum besseren Verständnis Ihrer Fähigkeiten und Einschränkungen haben wir einen Fragebogen ausgearbeitet, in dem Sie Ihren momentanen Zustand selbst einschätzen können. Damit helfen Sie uns, die Therapie Ihren persönlichen Bedürfnissen anzupassen. Ihre Angaben sind nur Ihrer Therapeutin bzw. Ihrem Therapeuten zugänglich und werden vertraulich behandelt.
Bitte bestimmen Sie selbst, wie weit die unten aufgeführten Aussagen für Sie übereinstimmen.
Bei allen Aktivitäten spielt es keine Rolle, ob Sie Hilfsmittel benützen oder nicht. Sie müssen es aber ohne Hilfe weiterer Personen ausführen können.
Bitte kreuzen Sie für jede Aussage und jede Frage die Zahl an, die am ehesten zutrifft.
Bei Unklarheiten wenden Sie sich bitte an Ihre Therapeutin bzw. Ihren Therapeuten.
Es gibt keine richtigen oder falschen Antworten sondern nur Ihre persönliche Einschätzung.
Herzlichen Dank für Ihre wertvolle Mitarbeit.

Hier ein Beispiel:

1. Ich kann ein Telefon bedienen. — (10) (20) (30) (40) (50) (60) (70) (80) (90)

Stimmt nicht — Stimmt teilweise — Stimmt genau

"10" bedeutet dabei "stimmt nicht",
"50" bedeutet "stimmt teilweise", und
"90" bedeutet "stimmt genau".

Diese Person ist eher nicht der Meinung, dass sie ein Telefon bedienen kann.

Bearbeiten Sie bitte jetzt folgende Fragen und Aussagen.

Version 7/01 © 2005, 2006, 2007 FPMS

FPMS
Fachgruppe Physiotherapie bei Multipler Sklerose

MS
Schweizerische Multiple Sklerose Gesellschaft

MSQPT®

Multiple Sclerosis Questionnaire for Physiotherapists Patienten-Fragebogen zur Therapieevaluation

Name des Patienten/der Patientin: ____________________

Physiotherapeut/Physiotherapeutin: ____________________

Physiotherapie seit: ____________________

Untersuchungsdatum: ____________________

Bitte beachten Sie, dass es keine Rolle spielt, ob Sie Hilfsmittel benützen oder nicht.
Sie müssen es aber ohne Hilfe weiterer Personen ausführen können.

Nr.	Frage	10	20	30	40	50	60	70	80	90
1	Im Vergleich zu der Situation vor einem halben Jahr, wie würden Sie Ihre Gesundheitssituation beschreiben?	*Viel schlechter*				*Gleich*				*Viel besser*
2	Wenn ich morgens aufwache, fühle ich mich ausgeruht.	*Stimmt nicht*				*Stimmt teilweise*				*Stimmt genau*
3	Ich kann mich ohne Hilfe anziehen.	*Stimmt nicht*				*Stimmt teilweise*				*Stimmt genau*
4	Ich kann selbstständig duschen.	*Stimmt nicht*				*Stimmt teilweise*				*Stimmt genau*
5	Ich kann selbstständig ein Bad nehmen.	*Stimmt nicht*				*Stimmt teilweise*				*Stimmt genau*
6	Ich habe Mühe beim Zähne putzen.	*Stimmt nicht*				*Stimmt teilweise*				*Stimmt genau*
7	Ich habe Schwierigkeiten sicher zu stehen.	*Stimmt nicht*				*Stimmt teilweise*				*Stimmt genau*

Version 7/01 © 2005, 2006, 2007 FPMS

8. A. Wie ***weit*** können Sie ohne Sitzpause auf flachem Boden gehen?

☐ 0 – 3 m	☐ 3 – 10 m	☐ 10 – 50 m	☐ 50 – 100 m	
☐ 100 – 500 m	☐ 500 – 1000 m	☐ 1 – 2 km	☐ 2 – 5 km	☐ über 5 km

B. Wie ***lange*** können Sie ohne Sitzpause auf flachem Boden gehen?

☐ 0 – 1 Min.	☐ 1 – 3 Min.	☐ 3 – 5 Min.	☐ 5 – 7 Min.	☐ 7 – 10 Min.
☐ 10 – 20 Min.	☐ 20 – 30 Min.	☐ 30 – 60 Min.	☐ 1 bis 2 Std.	☐ über 2 Std.

9 ▶ *Eine Treppe zwischen 2 Stockwerken hat 14 bis 16 Stufen.*
Bitte schätzen Sie, wie viele Stufen Sie ungefähr hinuntergehen können.

A. Wie viele Treppenstufen können Sie ***hinunter***gehen?

☐ 0 – 9	☐ 10 – 19	☐ 20 – 29	☐ 30 – 39	☐ 40 – 49
☐ 50 – 59	☐ 60 – 69	☐ 70 – 79	☐ 80 – 89	☐ über 90

B. Wie viele Treppenstufen können Sie ***hinauf***gehen?

☐ 0 – 9	☐ 10 – 19	☐ 20 – 29	☐ 30 – 39	☐ 40 – 49
☐ 50 – 59	☐ 60 – 69	☐ 70 – 79	☐ 80 – 89	☐ über 90

10 Ich kann selbstständig in ein Auto ein- bzw. aussteigen.

(10)–(20)–(30)–(40)–(50)–(60)–(70)–(80)–(90)
Stimmt nicht … *Stimmt teilweise* … *Stimmt genau*

11 Ich kann selbstständig öffentliche Verkehrsmittel benutzen.

(10)–(20)–(30)–(40)–(50)–(60)–(70)–(80)–(90)
Stimmt nicht … *Stimmt teilweise* … *Stimmt genau*

12 ▶ *Im Folgenden sind einige Tätigkeiten beschrieben, die Sie vielleicht an einem normalen Tag ausüben.*
Wie stark sind Sie durch Ihren derzeitigen Gesundheitszustand bei diesen Tätigkeiten eingeschränkt?

A. Wie stark sind Sie eingeschränkt bei anstrengenden Tätigkeiten wie z. B. schnell laufen, schwere Gegenstände heben, anstrengenden Sport treiben?

(10)–(20)–(30)–(40)–(50)–(60)–(70)–(80)–(90)
Nicht eingeschränkt … *Teilweise eingeschränkt* … *Stark eingeschränkt*

B. Wie stark sind Sie eingeschränkt bei mittelschweren Tätigkeiten wie z. B. einen Tisch verschieben oder staubsaugen.

(10)–(20)–(30)–(40)–(50)–(60)–(70)–(80)–(90)
Nicht eingeschränkt … *Teilweise eingeschränkt* … *Stark eingeschränkt*

C. Wie stark sind Sie eingeschränkt bei Einkaufstaschen heben oder tragen?

(10)–(20)–(30)–(40)–(50)–(60)–(70)–(80)–(90)
Nicht eingeschränkt … *Teilweise eingeschränkt* … *Stark eingeschränkt*

Version 7/01 © 2005, 2006, 2007 FPMS

13 Ich habe beim Schreiben ...

(10)–(20)–(30)–(40)–(50)–(60)–(70)–(80)–(90)
Keine Schwierigkeiten — *Mittlere Schwierigkeiten* — *Grosse Schwierigkeiten*

14 Ich fühle mich vital und habe Energie.

(10)–(20)–(30)–(40)–(50)–(60)–(70)–(80)–(90)
Stimmt nicht — *Stimmt teilweise* — *Stimmt genau*

Bitte beachten Sie, dass es keine Rolle spielt, ob Sie Hilfsmittel benützen oder nicht.
Sie müssen es aber ohne Hilfe weiterer Personen ausführen können.

15 Ich habe Probleme mit dem Gleichgewicht.

(10)–(20)–(30)–(40)–(50)–(60)–(70)–(80)–(90)
Stimmt nicht — *Stimmt teilweise* — *Stimmt genau*

16 A. Spastizität ist eines meiner Symptome.

☐ Ja → beantworten Sie bitte Frage 16 B.
☐ Nein

B. Ich kann die Spastizität beeinflussen.

▶ *Die Beeinflussung der Spastizität durch Medikamente ist bei dieser Frage ausgeschlossen.*

(10)–(20)–(30)–(40)–(50)–(60)–(70)–(80)–(90)
Stimmt nicht — *Stimmt teilweise* — *Stimmt genau*

17 Ich habe genügend körperliche Kraft, um meinen Alltag zu bewältigen.

(10)–(20)–(30)–(40)–(50)–(60)–(70)–(80)–(90)
Stimmt nicht — *Stimmt teilweise* — *Stimmt genau*

18 Wie gross ist Ihre Ermüdbarkeit?

(10)–(20)–(30)–(40)–(50)–(60)–(70)–(80)–(90)
Keine Ermüdbarkeit — *Mittelmässige Ermüdbarkeit* — *Sehr grosse Ermüdbarkeit*

19 Ich kann meine Ermüdung kontrollieren.

(10)–(20)–(30)–(40)–(50)–(60)–(70)–(80)–(90)
Stimmt nicht — *Stimmt teilweise* — *Stimmt genau*

20 Ich habe Schmerzen, die mich stören.

(10)–(20)–(30)–(40)–(50)–(60)–(70)–(80)–(90)
Stimmt nicht — *Stimmt teilweise* — *Stimmt genau*

Version 7/01 © 2005, 2006, 2007 FPMS

21 Gefühlsstörungen beeinträchtigen mich im Alltag.

▶ *Diese Frage bezieht sich auf körperliche Empfindungen.*

(10)—(20)—(30)—(40)—(50)—(60)—(70)—(80)—(90)
Stimmt nicht · *Stimmt teilweise* · *Stimmt genau*

22 Ich kann Aktivitäten ausüben, die ich gerne habe.

(10)—(20)—(30)—(40)—(50)—(60)—(70)—(80)—(90)
Stimmt nicht · *Stimmt teilweise* · *Stimmt genau*

23 Ich kann meine Aktivitäten gezielt einteilen, um meine Symptome der MS nicht zu verschlimmern.

(10)—(20)—(30)—(40)—(50)—(60)—(70)—(80)—(90)
Stimmt nicht · *Stimmt teilweise* · *Stimmt genau*

24 Die Blase macht mir Probleme.

(10)—(20)—(30)—(40)—(50)—(60)—(70)—(80)—(90)
Stimmt nicht · *Stimmt teilweise* · *Stimmt genau*

25 Ich habe Probleme mit dem Stuhlgang.

(10)—(20)—(30)—(40)—(50)—(60)—(70)—(80)—(90)
Stimmt nicht · *Stimmt teilweise* · *Stimmt genau*

26 Im Alltag fühle ich mich belastbar.

(10)—(20)—(30)—(40)—(50)—(60)—(70)—(80)—(90)
Stimmt nicht · *Stimmt teilweise* · *Stimmt genau*

27 Ich kann am Familienleben und im Freundeskreis aktiv teilnehmen.

(10)—(20)—(30)—(40)—(50)—(60)—(70)—(80)—(90)
Stimmt nicht · *Stimmt teilweise* · *Stimmt genau*

28 Ich kann einen Ausflug machen, der mich den ganzen Tag von zu Hause fern hält.

(10)—(20)—(30)—(40)—(50)—(60)—(70)—(80)—(90)
Stimmt nicht · *Stimmt teilweise* · *Stimmt genau*

29 Ich habe Angst vor möglichen Folgen der MS.

(10)—(20)—(30)—(40)—(50)—(60)—(70)—(80)—(90)
Stimmt nicht · *Stimmt teilweise* · *Stimmt genau*

30 Ich habe konkrete Ziele, an denen ich gerne arbeite.

(10)—(20)—(30)—(40)—(50)—(60)—(70)—(80)—(90)
Stimmt nicht · *Stimmt teilweise* · *Stimmt genau*

Herzlichen Dank!

Version 7/01 © 2005, 2006, 2007 FPMS
