# Supplementary material for: Patient-reported questionnaires in MS rehabilitation: responsiveness and minimal important difference of the multiple sclerosis questionnaire for physiotherapists (MSQPT)
Source: BMC Neurol. 2017 Mar 16;17:50. doi: 10.1186/s12883-017-0834-1 (PMC5356359; doi:10.1186/s12883-017-0834-1)
Supplement: Additional file 2: — Shows the English translation the items of the MSQPT. (PDF 98 kb) [file 12883_2017_834_MOESM2_ESM.pdf]

English translation of the items of the MSQPT

1	Compared to half a year ago, how would you describe your general health now?
2	When I wake up in the morning, I feel rested.
3	I can dress myself without assistance.
4	I can take a shower on my own.
5	I can take a bath on my own.
6	I have problems brushing my teeth.
7	I have problems standing safe.
8	A. How far can you walk on flat ground without sitting down?
	B. How far can you walk on flat ground without sitting down?
9	A. How many steps can you walk downstairs?
	B. How many steps can you walk upstairs?
10	I can get in and out of a car by myself.
11	I can use the public traffic by myself.
12	A. To what extend are you limited in strenuous activities like running, lifting heavy objects or doing an intensive work out?
	B To what extend are you limited in medium heavy activities like moving a table, hoovering, bowling or playing golf?
	C To what extend are you limited in lifting and carrying shopping bags?
13	I have difficulties in writing.
14	I feel vital and have energy.
15	I have problems with my balance.
16	Spasticity is one of my symptoms.
17	I have enough physical strength to manage my daily life.
18	How strong is your fatigue?
19	I can manage my fatigue.
20	I have pain that bothers me.
21	Disturbances of sensitivity affect my daily life.
22	I can engage myself in activities that I like.
23	I can grade my activities in a way that the symptoms of my MS do not get worse.
24	I have troubles with my bladder.
25	I have troubles with my defecation.
26	In my daily life, I feel myself resilient.
27	I can actively participate in family life and in my circle of friends.
28	I can go on a daily trip away from home.
29	I am afraid of the possible consequences of MS.
30	I have specific goals that I like to achieve.
